# Evidence for adaptive responses to historic drought across a native plant species range

**DOI:** 10.1111/eva.12803

**Published:** 2019-05-16

**Authors:** Erin E. Dickman, Lillie K. Pennington, Steven J. Franks, Jason P. Sexton

**Affiliations:** ^1^ Department of Life and Environmental Sciences University of California Merced California; ^2^ Yosemite National Park El Portal California; ^3^ Department of Biological Sciences Fordham University Bronx New York

**Keywords:** climate adaptation, drought, genetic variation, *Mimulus laciniatus*, postsown gibberellic acid treatment, resurrection study, species range limits

## Abstract

As climatic conditions change, species will be forced to move or adapt to avoid extinction. Exacerbated by ongoing climate change, California recently experienced a severe and exceptional drought from 2011 to 2017. To investigate whether an adaptive response occurred during this event, we conducted a “resurrection” study of the cutleaf monkeyflower (*Mimulus laciniatus*), an annual plant, by comparing trait means and variances of ancestral seed collections (“pre‐drought”) with contemporary descendant collections (“drought”). Plants were grown under common conditions to test whether this geographically restricted species has the capacity to respond evolutionarily to climate stress across its range. We examined if traits shifted in response to the recent, severe drought and included populations across an elevation gradient, including populations at the low‐ and high‐elevation edges of the species range. We found that time to seedling emergence in the drought generation was significantly earlier than in the pre‐drought generation, a response consistent with drought adaptation. Additionally, trait variation in days to emergence was reduced in the drought generation, which suggests selection or bottleneck events. Days to first flower increased significantly by elevation, consistent with climate adaptation across the species range. Drought generation plants were larger and had greater reproduction, which was likely a carryover effect of earlier germination. These results demonstrate that rapid shifts in trait means and variances consistent with climate adaptation are occurring within populations, including peripheral populations at warm and cold climate limits, of a plant species with a relatively restricted range that has so far not shifted its elevation distribution during contemporary climate change. Thus, rapid evolution may mitigate, at least temporarily, range shifts under global climate change. This study highlights the need for better understanding rapid adaptation as a means for plant communities to cope with extraordinary climate events.

## 1 INTRODUCTION

Global climate change presents a serious and immediate threat to ecosystem structure and function (Loarie et al., 2009; Sala et al., 2000), and the current rates of climate change are unprecedented (Diffenbaugh & Field, 2013). Under changing climates, species will be forced to move or adapt to avoid extinction, with some studies already documenting climate‐driven declines in biodiversity (Harrison, Gornish, & Copeland, 2015; Martay et al., 2017; Wernberg et al., 2011).

Plant responses to climatic change, such as range shifts (Kopp & Cleland, 2014; Parmesan & Yohe, 2003; Root et al., 2003; Walther et al., 2002; Wolf, Zimmerman, Anderegg, Busby, & Christensen, 2016) and adaptation (Franks, 2011; Franks, Sim, & Weis, 2007; Hairston et al., 1999; Parmesan, 2006; Sultan, Horgan‐Kobelski, Nichols, Riggs, & Waples, 2013), can be rapid. However, little is known about how climate change affects populations across their range, especially at their range limits. In particular, the extremes of a species range (i.e., elevation, latitude) are important to understand as they are where range expansion or contraction may occur (Hampe & Petit, 2005). The lowest elevation populations, the potential “rear edge or trailing edge,” may face the warmest and driest conditions. These populations may exhibit local extirpation and may be disproportionally affected by climate change, resulting in range contraction (Aitken, Yeaman, Holliday, Wang, & Curtis‐McLane, 2008; Bertrand et al., 2011; Bridle & Vines, 2007; Hampe & Petit, 2005; Sexton, Strauss, & Rice, 2011). Range‐restricted or endemic species may be particularly vulnerable as they are at higher risk of extinction (Dirnböck, Essl, & Rabitsch, 2011; Parmesan, 2006; Pimm & Raven, 2000).

Vulnerability to climate shifts is related to the amount of genetic variation present for natural selection to act upon in a population. Populations at species range limits may be smaller in size and lack sufficient genetic variation to respond to changing climates (Dawson, Grosberg, Stuart, & Sanford, 2010; Holt, Gomulkiewicz, & Barfield, 2003; Kirkpatrick & Barton, 1997). Alternatively, populations at species range limits may have substantial genetic variation (Holt & Gomulkiewicz, 1997; Sexton et al., 2011) and may already have some degree of local climate adaptation that could provide critical genetic variation to other populations within the species' range (Hampe & Petit, 2005; Holt & Gomulkiewicz, 1997; Macdonald, Llewelyn, Moritz, & Phillips, 2017; Sexton et al., 2011).

A critical factor of species' responses to climate stress is timing their developmental stages to maximize limited resources and increase their chance of survival to reproduce (Cleland, Chuine, Menzel, Mooney, & Schwartz, 2007; Dijk & Hautekèete, 2014; Kimball, Angert, Huxman, & Venable, 2010; Thomann, Imbert, Engstrand, & Cheptou, 2015). Selection for faster development and/or earlier flowering due to elevated CO_2_ (Springer & Ward, 2007), dry soil (Ivey & Carr, 2012), and reduction in precipitation (Franks et al., 2007) has been documented in some plant species and can facilitate drought escape in shortened growing seasons. Critical photoperiod is the primary control over phenology in temperate climates, with temperature as a secondary moderating effect (Körner & Basler, 2010). Photoperiod is not affected by climate, and as snowpack declines and peak runoff dates shift earlier in the growing season, there could be a mismatch between germination cues and resource availability, leading to reduced fitness (Anderson, Inouye, McKinney, Colautti, & Mitchell‐Olds, 2012). Thus, reduced sensitivity to photoperiod has been shown to be adaptive with changes to climate and can result in faster flowering and shorter seed dormancy (Franks & Hoffmann, 2012).

The “resurrection” approach has recently emerged to document trait shifts (e.g., phenology) due to contemporary evolution (Dijk & Hautekèete, 2014; Franks et al., 2008, 2007; Franks, Hamann, & Weis, 2018; Hairston et al., 1999; Kuester, Wilson, Chang, & Baucom, 2016; Sultan et al., 2013). This approach takes ancestral and descendent seeds collected from a population and raises them in a common environment. Differences in phenotype between ancestors and descendants provide evidence of evolutionary change that has taken place in the interval between the two collections. The resurrection approach is a powerful tool for analyzing contemporary evolutionary responses to changes in climate (Franks et al., 2018).

One area that has experienced very substantial changes in climatic conditions, and extremes in climatic fluctuations over the last few decades, is the region of southern and central California. The California Sierra Nevada has a Mediterranean climate characterized by cool, wet winters and warm dry summers. The state's climate, particularly its precipitation, is variable year to year and features wider swings between wet and dry years than in any other state in the United States (Barbour, Keeler‐Wolf, & Schoenherr, 2007; Dettinger et al., 2011). Exacerbated by the global trend of hotter and drier climates, California recently experienced an exceptional drought beginning in 2011 and containing the driest 12‐month period on record between 2013 and 2014 (Swain et al., 2014). The effects of the water deficit have been magnified by record high temperatures (Griffin & Anchukaitis, 2014). Moreover, the drought in 2014 has an estimated return interval of 700–900 years, and the cumulative drought of 2012–2014 has an estimated return interval of over 1,200 years (Robeson, 2015). The Sierra Nevada is home to a great diversity of endemic species living along its steep elevational gradients, and climate change is having dramatic effects on these and other regional ecosystems (Harrison et al., 2015; Kelly & Goulden, 2008; Kimball et al., 2010; McIntyre et al., 2015; Moritz et al., 2008). However, the adaptive capacity of native populations in these systems is virtually unknown.

To investigate the effect of the recent, severe drought on the adaptive response of plants across their species range, we conducted a resurrection study of the Sierra endemic, cutleaf monkeyflower, *Mimulus laciniatus* A. Gray. We asked the following questions: (a) Have traits shifted in response to the recent, severe drought? and (b) If there are trait shifts, do shifts depend on elevation? Previous studies have found evidence for evolved, earlier development in response to drought that translated into greater fitness under drought conditions (e.g., earlier flowering in *Brassica*, Franks et al., 2007). We compared phenological and morphological trait values of ancestor and descendant seed collections, collected at two separate years at the same populations. We grew seeds in a greenhouse under common conditions from nine populations across the species range, including its elevational extremes representing the potential leading and rear edge. Ancestors (hereafter referred to as “pre‐drought generation”) were collected in years with typical precipitation in 2008 or earlier, and descendants (hereafter referred to as “drought generation”) were collected in an exceptional drought year, 2014. *Mimulus laciniatus* is a highly self‐fertilizing annual plant. In this resurrection study, we report first‐generation responses that include broad‐sense heritabilities, which are fundamental to adaptive potential in highly selfing species and which apply to a substantial proportion of flowering plants (Goodwillie, Kalisz, & Eckert, 2005). We confirmed that phenology differences between drought and pre‐drought generations likely had a genetic basis by observing seed emergence in a subsequent generation. We hypothesized that under extreme drought conditions, given sufficient variation, plant populations should shift their phenotypes toward more drought‐adaptive strategies (Franks et al., 2007).

## 2 MATERIALS AND METHODS

### 2.1 Study system

*Mimulus laciniatus* is an annual, herbaceous plant endemic to the western slope of the central Sierra Nevada and limited in its distribution due to its habitat requirements (Sexton & Dickman, 2016). It primarily inhabits snowmelt seeps and moss patches on granite outcrops between ca. 900 and 3,270 m, many of which progressively dry during the growing season. *Mimulus laciniatus* spans several biotic zones in the Sierra Nevada, including the foothill woodland, the montane mixed‐conifer, and the subalpine and alpine communities (Sexton et al., 2016). It is a winter annual that germinates during the late fall and winter rains characteristic of its Mediterranean climate (Cowling, Rundel, Lamont, Kalin Arroyo, & Arianoutsou, 1996). It develops a small basal rosette of leaves through the winter, flowers during the spring or early summer and senesces in the dry late spring or summer depending on elevation. It is primarily self‐pollinating (roughly 95%; Ferris, Sexton, & Willis, 2014), though it can be visited by bees and other insects (Sexton et al., 2011). Since *M. laciniatus* is largely self‐pollinating, maternal and epigenetic effects may be important components of its adaptive response for coping with environmental stress (Germain, Caruso, & Maherali, 2013).

**Figure 1:**
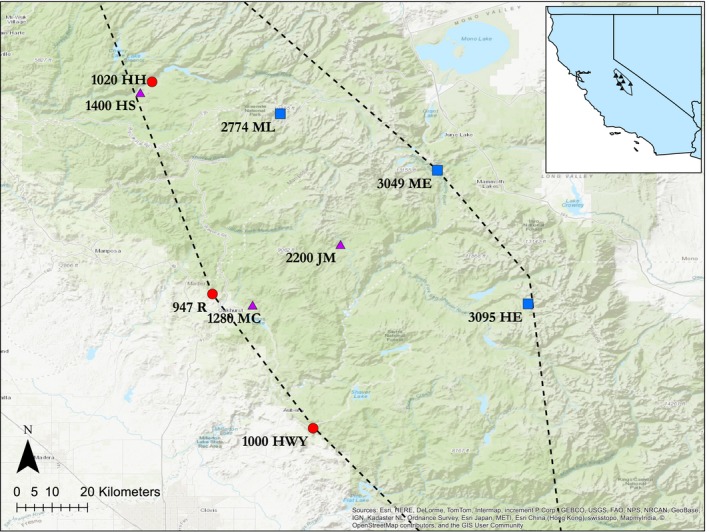
Map of study locations. Black dotted line indicates extent of *Mimulus laciniatus* species range. The red circles denote the three lowest elevation populations located at the low edge of the species range, labeled R, HWY, and HH. The purple triangles denote the three intermediate‐elevation populations of the species range, labeled MC, HS, and JM. The blue squares denote the three highest elevation populations at the high edge of the species range, labeled ML, ME, and HE. Numbers before labels are elevation in meters. Inset map shows location of study populations within the central portion of the Sierra Nevada, California

We collected seeds from nine *M. laciniatus* populations at two periods in time (Table S1). Seeds were collected randomly within each population to maximize genetic diversity related to habitat heterogeneity (following Sexton et al., 2016). Pre‐drought generation seeds were collected in 2006 for all populations with the exception of Hwy 168 (HWY) and Hetchy Sign (HS), which were collected in 2005, and Jackass Meadow (JM), which was collected in 2008. The drought generation seeds were collected in 2014 for all populations. The nine localities span a wide set of heterogeneous habitats and elevations, from the lowest at 947 m to the highest at 3,095 m, and represent the entirety of the species elevational range. Of these, three populations were sampled near low‐elevation extremes; three from high‐elevation extremes; and three from more intermediate elevations. These populations are located within Yosemite National Park, Sierra National Forest, and private property (Figure 1).

### 2.2 Greenhouse experiment

To assess seed viability, we conducted cut tests of seeds from 30 randomly drawn maternal families from pre‐drought and drought generations (Ooi, Auld, & Whelan, 2004). All seeds were examined under a dissecting microscope and appeared to have a normal endosperm and a live embryo, which indicates viability (Baskin & Baskin, 2014; Bonner & Russell, 1974).

We planted field‐collected seeds from 30 maternal families per site, for each of the pre‐drought and drought generations. As the drought generation experienced an extreme climate and had low seed yield, there were two populations, May Lake (ML) and Mammoth Edge (ME), from which we could not obtain 30 maternal families. We planted 12 maternal families for ML and 14 maternal families for ME for a total of 510 maternal families for the experiment. For one site, ME, no field‐collected seeds were available in the pre‐drought generation. However, seeds from plants that had been self‐pollinated for one generation in the greenhouse after collection during the pre‐drought period at ME were available, and these were used as pre‐drought seeds for ME in our experiment.

Seeds were randomly sown into Sunshine Mix #1 potting soil (Sun Gro Horticulture) in eight trays with 72‐cell, black, plastic planters using a randomized block design. Ten seeds from each maternal family were sown into a cell, except in rare cases where fewer were available. After sowing, we added 1cm of sand mulch to the top of each cell, filled the tray bottom with water, covered the tray with a black plastic lid, and placed trays in a 4°C vernalization cabinet for 11 days (Friedman & Willis, 2013). After vernalization, we moved trays to a greenhouse, where plants received natural light and moderate ambient temperatures between 18.5 and 30.1°C. Trays were filled with reverse osmosis water as needed to maintain saturated soil. Once per week, they received a nutrient mix water that contained a 1.3% concentration of fertilizer (Grow More Inc.), magnesium sulfate, and calcium nitrate.

Plants were surveyed weekly for phenology and morphology traits. Once a seedling was growing in a cell, the individual closest to the center was selected and the other seedlings were documented and thinned. Phenology was recorded as the most advanced stage on the plant: (a) seedling (emerged from soil, vegetative), (b) budding (flower buds present), (c) flowering (at least one open flower was present), (d) fruiting (at least one fruit was present), or (e) dead (dry, senesced; Franks et al., 2007; Jonas & Geber, 1999; Schneider & Mazer, 2016). Using these data, we calculated days to emergence, defined as the first day when a plant was observed in a cell; days to flower, defined as the first day a flower is observed on a plant; and days to first flower, defined as the number of days between emergence and flowering (Franks et al., 2007; Jonas & Geber, 1999; Schneider & Mazer, 2016). There were some instances when a plant recorded as “bud” 1 week had a mixture of fruits and flowers the next. In such instances, the stage was entered as “flower.” We also measured traits related to growth, resource allocation, and drought response, including height and specific leaf area (SLA; Ackerly, Knight, Weiss, Barton, & Starmer, 2002; Dolph & Dilcher, 1980; Mooney & Dunn, 1970; Ostertag, Warman, Cordell, & Vitousek, 2015; Peñuelas & Matamala, 1990). For SLA, one basal leaf was collected from the most basal node when a plant was fruiting and photographed, dried, and weighed.

After 105 days of the experiment, 36.86% of the cells had plants, which were largely senescing. This left 63.14% of maternal families planted that had not germinated. It is possible that photoperiod or temperatures were not ideal in the greenhouse in the early spring to promote germination for all populations or that the 11‐day vernalization period was not sufficient for all populations. To test seed viability and confirm dormancy of those that did not germinate, we exposed these cells to two experimental postsown treatments. We moved all living plants from their cells and transplanted them to new, identical trays. The original, untreated trays then contained only cells that had not germinated. Half of trays (178 cells) received a gibberellic acid solution (5 ml per cell of 200 ppm concentration) applied to the soil surface and 24 hr later were rinsed with running water for 3 min. The other half of trays (172 cells) were returned to the 4°C vernalization chamber for 6 days with darkness, and then seven more days with light, and were subsequently returned to the greenhouse. The plants that grew initially, prior to the gibberellic acid or second vernalization, will hereafter be referred to as “untreated” group; those receiving the gibberellic acid will be referred to as “GA” group; and those receiving the second vernalization will be referred to as “vernalized” group. The GA and vernalized plants were treated and returned to the greenhouse in early June (June 2 and 15, respectively). Plants were allowed to grow for 7 months at which time the majority (81%) had senesced. We ended the experiment on September 25. Plants that had not senesced included individuals that were still vegetative (there may have been an inadequate photoperiod to cue flowering in these individuals by the beginning of fall) or reproductive but not yet senescent. After accounting for mortality, we recorded data for 398 individuals, each representing a unique maternal family.

Plants were clipped at the soil surface, excluding roots. Total number of fruits were counted, removed, collected, and weighed. Fruit mass was used as a proxy for fitness, as in Sexton et al. (2011). Nonreproductive aboveground biomass was placed into a drying oven at 60°C for 48 hr and then weighed. The weight of the single leaf harvested from each plant for the SLA analysis was added to the total. To estimate SLA, leaf photos were processed using Image J software to obtain leaf area (Schneider, Rasband, & Eliceiri, 2012).

### 2.3 Growth chamber experiment

Since emergence timing varied in important ways that subsequently affected plant fitness between drought and pre‐drought generations (see Phenotypic evolution in response to drought section in Results), we raised all descendants for an additional generation within growth chambers. (These chambers became available only after the greenhouse experiment was concluded). In this “confirmatory” generation, seeds were sown into a randomized block design and cold stratified in darkness for 2 weeks at 4°C. Trays were then moved to growth chambers and grown with a 16‐hr, 500 μmol light day with a daytime maximum of 25°C ramping down to 10°C at night. Plants were checked daily for emergence for 3 weeks.

### 2.4 Accounting for maternal effects on seed mass

Maternal effects (also referred to as “transgenerational effects”) on seed quality can affect subsequent phenotypic traits in common gardens (Heger, Jacobs, Latimer, Kollmann, & Rice, 2014; Roach & Wulff, 1987) and can act as important adaptive mechanisms in the wild (Galloway & Etterson, 2007; Germain et al., 2013). Maternal effects can also be important and inseparable components of phenotypic genetic variance, especially for a highly selfing species such as *M. laciniatus*. To account for potential maternal effects driven by seed mass differences, we estimated mean seed mass for a subset of maternal families that had ample seeds (406% or 79.6% of maternal families planted). *Mimulus laciniatus* seeds are tiny (generally <1 mm), and so we calculated mean seed mass by weighing 10–30 field‐collected seeds per family. Mean seed mass was included as a covariate in statistical models to account for potential maternal effects (Jonas & Geber, 1999; Schneider & Mazer, 2016).

### 2.5 Climate data

To estimate and compare climate trends, we obtained data for each population, extrapolated from the United States Geologic Survey Basin Characterization Model (270 m resolution; Flint & Flint, 2014). We obtained water year data for the year of seed collection at each population from the pre‐drought and drought collection years. We used the United States Geologic Survey definition of water year, defined as the period from October 1 of the previous year to September 30 of the current year (United States Geological Survey, 2016). Using water year data, rather than calendar year, is preferable because it includes the fall through spring, when the Sierra Nevada receives the majority of its precipitation and represents the conditions under which seeds germinate, grow, and reproduce. We obtained climatic water deficit (CWD; mm), total water year precipitation (mm), and mean maximum annual temperature (Tmax, °C). CWD is defined as the evaporative demand exceeding available soil moisture, calculated by subtracting actual evapotranspiration from potential evapotranspiration (Flint & Flint, 2014). We also obtained 30‐year annual averages (1981–2010) for precipitation and the temperature maximum and minimum for each population. We imported these data into R Version 0.99.903 (R Core Team, 2016) and calculated precipitation and temperature anomaly by subtracting the 30‐year annual water year average from values of the water year of seed collection to obtain a departure from climate normals. Since plants tend to be locally adapted, largely driven by climate (Clausen, Keck, & Hiesey, 1941; Hereford, Elle, & Geber, 2009; Leimu & Fischer, 2008), understanding the magnitude of extreme climate events relative to climate averages can help frame and direct studies of climate change response and adaptation.

### 2.6 Statistical analysis

To detect differences in phenological traits (i.e., days to emergence and first flower), between generations and among populations occupying different elevations, we conducted survival analyses using Cox Proportional Hazards models (Fox, 2001). These analyses can accept censored values, which in this experiment were individuals that never emerged when testing time to emergence and individuals that emerged but never flowered when testing differences in days to first flower. Due to the disruption in timing of the GA and vernalization treatments (e.g., most of the GA‐treated plants emerged simultaneously), only the untreated cohort is included in phenological analyses for the greenhouse experiment (see Germination section in Results). For the second generation in the growth chamber experiment, all seeds were included, and cohort was included in the model to control for cohort effects. We fit models for response variables days to emergence, days to flower, and days to first flower; we included generation (pre‐drought or drought), elevation (covariate), elevation by generation interaction, cohort, and mean seed mass (covariate) as explanatory variables; tray and population were included as random effects. Significance of explanatory variables was tested using likelihood ratio tests. The survival analyses were conducted in R Version 0.99.903 (R Core Team, 2016) using the coxme package (Therneau, 2018).

For analyses of morphological traits, all variables were transformed using average ranks (Conover & Iman, 1981) because standard transformations (i.e., log, square root, box cox, etc.) did not sufficiently meet the assumptions of parametric analyses. We created a Pearson correlation matrix in R using the Hmisc package (Harrell & Dupont, 2016) to examine whether any traits are highly correlated. We used a REML model (Shaw, 1987) with total plant mass, fruit mass, vegetative biomass, number of fruits, plant height, and SLA as response variables; generation (pre‐drought or drought), elevation (covariate), elevation by generation interaction, germination cohort (untreated, GA, or vernalized), and mean seed mass (covariate) as explanatory variables; tray and population were included as random variables. REML analyses were conducted in JMP**^®^** Pro (Version 12.0.1. SAS Institute Inc., 1989–2007) and were restricted to plants that emerged.

Finally, we conducted Levene's tests of homogeneity of variance in R (R Core Team, 2016), using the car package (Fox & Weisberg, 2011), to determine whether trait variance differed by generation or population. For a highly selfing plant like *M. laciniatus*, variance among full‐sibling families (i.e., genetic lineages) is the most relevant measure of genetic variance (Conner & Hartl, 2004). Thus, we used population trait variance as a proxy for trait genetic variance. We also calculated the coefficient of variation (CV) for each trait using the raster package (Hijmans & van Etten, 2012) to estimate trait variance among maternal families. These data were used as a proxy to compare genetic variation among populations and whether variance was reduced during the drought of 2012–2014.

## 3 RESULTS

### 3.1 Climatic variation over time

There was a substantial decline in available soil moisture over the period of the study, with a change from average conditions to severe drought. The climate leading up to the year of collection for the pre‐drought generation was wetter than average (Table 1). In contrast, the climate leading up to the year of collection for the drought generation was exceptionally dry and hot as compared to averages, across all elevations. To focus on the climate that produced seeds in the field, we report differences between the generations for the water year of seed collection (Table 1).

**Table 1:** BCM model climate values for the water year for all study populations in pre‐drought and drought seed collection years (Ppt is precipitation; Tmax is maximum temperature)

Population and elevation (m)	Climatic water deficit (mm)	Total water year precip. (mm)	Percent precip. deviation from 30‐year mean	Mean annual Tmax (°C)	Percent Tmax deviation from 30‐year mean	30‐year mean annual ppt (mm)	30‐year mean Tmax (°C)
R (947)							
Pre‐drought	890.15	1,109.58	0.33	22.34	0.00	834.47	22.26
Drought	1,037.98	394.79	−0.53	23.87	0.07		
HWY (1,000)							
Pre‐drought	508.84	1,054.68	0.41	20.44	−0.03	749.66	21.17
Drought	1,108.27	351.50	−0.53	22.69	0.07		
HH (1,020)							
Pre‐drought	671.34	1,303.89	0.38	19.33	0.02	947.43	19.01
Drought	757.93	511.51	−0.46	21.77	0.15		
MC (1,280)							
Pre‐drought	886.05	1,370.47	0.36	19.82	0.00	1,009.46	19.74
Drought	954.82	458.27	−0.55	21.86	0.11		
HS (1,400)							
Pre‐drought	525.47	1,353.10	0.43	17.58	−0.04	946.39	18.30
Drought	750.78	544.24	−0.42	19.67	0.07		
JM (2,200)							
Pre‐drought	775.54	781.88	−0.32	14.58	0.02	1,149.20	14.23
Drought	777.78	521.05	−0.55	16.41	0.15		
ML (2,774)							
Pre‐drought	166.83	1,985.16	0.43	11.05	0.02	1,392.92	10.86
Drought	424.82	616.10	−0.56	12.29	0.13		
ME (3,049)							
Pre‐drought	294.09	1,066.41	0.35	9.20	−0.01	790.03	9.26
Drought	386.82	501.27	−0.37	11.39	0.23		
HE (3,095)							
Pre‐drought	272.93	1,372.34	0.37	8.43	−0.02	1,003.11	8.60
Drought	351.34	542.81	−0.46	10.40	0.21		

Moisture stress was higher for all drought generation populations. Drought generation populations had greater CWD than pre‐drought generation populations, but populations varied greatly in their degree of change (Figure S1). Taking CWD values from Table 1 and calculating percent change in CWD (i.e., subtracting drought generation CWD from pre‐drought generation CWD and dividing the difference by pre‐drought CWD), populations varied from 0.3% change (JM) to 155% change (ML). The greatest increase of 155% at the high population ML was followed by 118% at the low population HWY. This increase in moisture stress was driven by a combination of very low precipitation and high temperatures. Total water year precipitation was lower for drought generation populations than pre‐drought generation populations. All pre‐drought generation populations had increased precipitation relative to the 30‐year mean at that locality, with the exception of population JM; however, those seeds were collected in 2008 (Table 1). Drought generation populations all had decreased precipitation relative to the 30‐year mean, ranging from 36.6% to 55.8% reductions in precipitation (Table 1). Maximum temperature (Tmax) was higher for all drought generation populations than pre‐drought generation populations. Drought generation population Tmax anomaly all demonstrated increases from the 30‐year means, from a minimum increase of 1.4°C for population ML to a maximum increase of 2.8°C for population HH (Table 1).

### 3.2 Germination

Germination varied greatly among treatments and populations. Of the untreated group, the three highest elevation populations had lowest germination (17.3%). In contrast, the six lower and intermediate elevation populations had very similar germination (43.3% and 45.0%, respectively; Table S2). Despite these elevational differences, almost all untreated plants that germinated flowered (95.0%). The GA group had the highest germination (94.9% of individuals treated) but only a little over half flowered (54.4%). Of those that flowered, 61% were from the pre‐drought group and 39% were from the drought group. The vernalized group had the lowest germination (26.0% of individuals treated) and the lowest flowering rate (38.6%).

### 3.3 Phenotypic evolution in response to drought

We found significant evolutionary change in several traits and in trait variances. These evolutionary changes were shown by differences between ancestors and descendants grown under common conditions. Regarding phenology, days to emergence differed significantly by generation; mean day of emergence was 4.4 days earlier for the drought generation than the pre‐drought generation (Table 2). Emergence time significantly decreased with elevation, but the interaction between elevation and generation was not significant. The mean seed mass covariate was significant (Table 2).

**Table 2:** Cox proportional hazards model results for phenological data

Explanatory variable	Days to emergence			Days to first flower		
	*df*	*X* ^2^	*p* value	*df*	*X* ^2^	*p* value
Elevation	10.17	0.36	<**0.001**	11.26	0.71	<**0.001**
Generation	8.73	53.15	<**0.001**	9.36	60.76	<**0.001**
Elevation × Generation	9.61	0.87	0.351	10.81	1.47	0.225
Mean seed mass	11.65	296.84	<**0.001**	11.64	276.66	<**0.001**

Note

Values in bold were significant at *α* = 0.05.

**Figure 2:**
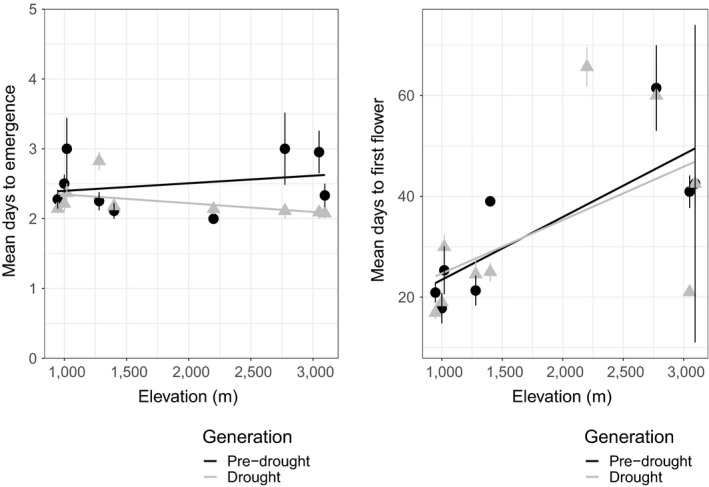
Days to emergence for second‐generation seedlings and days to first flower for first‐generation plants by elevation and pre‐drought/drought generation. Vertical bars represent 1 standard error. Regression trend lines on population means across elevation are plotted for reference only

In the next generation grown within growth chambers, drought generation seeds maintained earlier emergence, emerging an average of 0.3 days earlier than the pre‐drought generation (Figure 2). Seeds had higher and much faster germination rates within growth chamber conditions; 98% and 89% of seeds germinated from the pre‐drought and drought generations, respectively, and 93% of seeds germinated within nine days. Generation (*df* = 16.03, *X* ^2^ = 9.51, *p* = 0.009) was significant, whereas elevation (*df* = 17.04, *X* ^2^ = 2.57, *p* = 0.12), the interaction between generation and elevation (*df* = 17.22, *X* ^2^ = 2.60, *p* = 0.12), and cohort (*df* = 15.65, *X* ^2^ = 3.19, *p* = 0.20) were not.

Levene's tests of equality of variances for days to emergence provided evidence that family‐based genetic variation was reduced in the drought generation. Plants differed significantly in variance by generation (Table S3), with a lower CV for the drought generation than the pre‐drought generation (Table S4). Levene's test for populations was marginally significant (Table S3).

Days to first flower significantly differed by generation. Mean days to first flower, postemergence, in the drought generation was 2.9 days longer, relative to the pre‐drought generation (Table 2, Figure 2). Days to first flower differed significantly by elevation (Table 2, Figure 2), and the pattern of variation suggests elevation‐based climate adaptation, with flowering speed decreasing with elevation (Figure 2). The interaction between generation and elevation was not significant, whereas the mean seed mass covariate was significant (Table 2, Figure 2). Levene's tests for days to first flower did not differ significantly by population or generation (Table S3). Results for days to flower and days to first flower (see Methods) were qualitatively similar (i.e., the same effects were significant, and responses varied consistently among treatments) for all analyses, and thus, days to flower data are not presented here.

**Figure 3:**
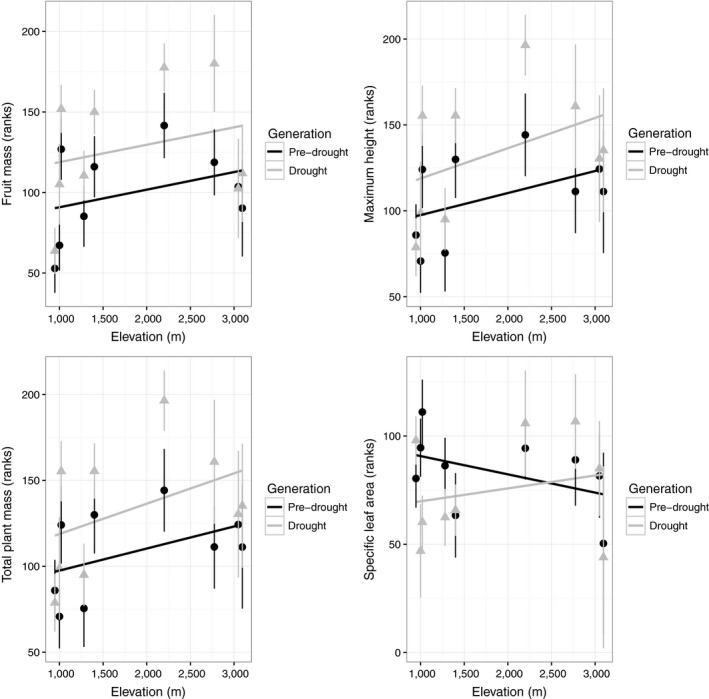
Morphological traits by elevation and pre‐drought/drought generation. Vertical bars represent 1 standard error. Regression trend lines on population means across elevation are plotted for reference only

Regarding morphological traits, drought generation plants were generally larger, taller, and had greater reproduction than pre‐drought generation plants. Mean fruit mass, total plant mass, and maximum height differed significantly by generation (Table 3, Figure 3). Trait variances did not differ significantly by generation or population for fruit mass. Total plant mass and maximum height variance differed significantly between populations, but not generations (Table S3). Fruit mass, total mass, height, and SLA did not differ significantly by elevation (Table 3, Figure 3). No significant interactions between generation and elevation nor mean seed mass covariate effects were found for morphological traits, whereas cohort effects (germination treatments) were significant for all morphological traits (Table 3). The Pearson correlation matrix revealed that two pairs of traits were highly correlated (nonreproductive biomass and total mass, *r* = 0.971, *p* < 0.0001; number of fruits and fruit mass, *r* = 0.835, *p* < 0.012). Thus, we do not present model results for nonreproductive biomass and number of fruits.

**Table 3:** Mixed REML model results for morphological traits

Response variable	*df*	*df* Den	*F* Ratio	*p* value
Fruit mass				
Generation	1	228.1	10.33	**0.002**
Elevation	1	7.9	1.12	0.322
Generation × Elevation	1	228.8	0.16	0.692
Cohort	2	185.8	69.40	**<0.001**
Mean seed mass	1	228.1	0.40	0.530
Total plant mass				
Generation	1	230.2	5.85	**0.016**
Elevation	1	8.3	2.40	0.158
Generation × Elevation	1	229.8	0.95	0.330
Cohort	2	159.4	18.45	**<0.001**
Mean seed mass	1	230.7	0.12	0.726
Maximum height				
Generation	1	285.3	5.22	**0.023**
Elevation	1	8.2	0.00	0.955
Generation × Elevation	1	285.2	0.05	0.819
Cohort	2	224.6	37.38	**<0.001**
Mean seed mass	1	285.6	0.77	0.381
Specific leaf area				
Generation	1	142.9	1.92	0.168
Elevation	1	24.03	0.02	0.880
Generation × Elevation	1	138.3	1.29	0.258
Cohort	2	59.98	23.86	**<0.001**
Mean seed mass	1	132.9	0.41	0.522

Note

Values in bold were significant at *α* = 0.05.

## 4 DISCUSSION

These results demonstrate that populations of a native plant with a restricted range are capable of responding to severe drought within a few years. Previous studies have demonstrated rapid evolution in plant populations in response to environmental changes, and many of these studies included weedy or introduced species with broad geographic ranges (e.g., Franks, 2011; Franks et al., 2007; Kuester et al., 2016; Parmesan, 2006; Sultan et al., 2013; Thomann et al., 2015), which can benefit from increased genetic variation from population mixing or hybridization during the invasion process (e.g., Gaskin & Schaal, 2002; Lavergne & Molofsky, 2007). In contrast, peripheral populations and species with restricted ranges have been viewed as potentially unable to respond quickly or effectively to a strong selective pressure due to lack of genetic variation (Dawson et al., 2010; Holt et al., 2003; Kirkpatrick & Barton, 1997; Pujol & Pannell, 2008; but see Sheth & Angert, 2016).

### 4.1 Rapid, contemporary evolution

Using the resurrection approach, we found compelling evidence that phenological traits shifted in an adaptive manner during the intense drought, and this may partially explain how the species range of this plant has remained stable over recent decades (Sexton & Dickman, 2016), whereas other species are exhibiting range shifts due to severe drought and climate change (Crockett & Westerling, 2017; Serra‐Diaz et al., 2015). We documented a significant reduction in time to emergence that would be adaptive in hotter and drier climates, accompanied by a reduction in variance in emergence time in the drought generation. Drought generation plants generally emerged earlier, and subsequently achieved greater height, biomass, and fruit mass during the experiment. There was no difference between generations in SLA (discussed below), and in contrast to emergence patterns, drought generation plants generally flowered later than pre‐drought plants and did not show differences in variance between generations in these traits. Nevertheless, flowering time is a strongly differentiated trait among populations (discussed below).

Two lines of evidence suggest that the observed differences in the drought generation may have been adaptive. First, the faster seed emergence observed in the drought generation is consistent with field experiments with this species in which earlier emergence in seedlings translated into greater fitness under drought stress (Sexton et al., 2011). The evolutionary changes in phenology did not vary by elevation (i.e., no significant generation by elevation interaction), although populations varied greatly in their responses across the range (Figure 2).

Second, variance in some phenological and morphological traits was reduced in the drought generation, suggesting natural selection or bottleneck events may have occurred. Rapid increases in temperature have been shown to be associated with a reduction in genetic variation for traits affected by climate (Jump & Peñuelas, 2005). With the exception of days to first flower, all traits had reduced variance in the drought generation (Table S4); however, only days to emergence exhibited significant variance reduction (Table S3). By comparison, SLA, an important trait for drought tolerance but not necessarily drought avoidance (Ackerly et al., 2002), was not found to vary significantly by population or generation. However, drought generation plants generally flowered later, opposite to emergence responses, suggesting that flowering time may have been under selection, but in a direction consistent with greater drought tolerance rather than avoidance. Early flowering is indicative of drought escape in annual plants because early flowering allows plants to complete their life cycle before the onset of drought. However, late flowering might potentially indicate drought tolerance or at least a strategy of growing more slowly and conserving resources during drought. Franks (2011) found that *Brassica rapa* plants that flowered early had low water use efficiency, whereas plants that flowered later had greater water use efficiency, indicating greater drought tolerance.

The above changes in phenotypic variance might potentially reflect changes in additive genetic variance, since genetic variance and phenotypic variance are often related, but this was not possible to determine from this study. A change in phenotypic variance is still notable, since this is a change in the population and one that could potentially influence the results of future selection. Reductions in trait variance could also be due to bottlenecks (i.e., genetic drift) and due to reduced population sizes under drought conditions. However, if the observed changes were due mainly to genetic drift, we would predict random trait mean shifts in adaptive and nonadaptive directions and reductions in variance across all or most traits. Instead, variance reductions align with trait shifts related to drought avoidance and are generally consistent among populations. Although the resurrection approach alone only provides evidence for evolution, rather than providing the mechanism (Franks et al., 2018), the idea that this phenotypic shift was caused by selection rather than by drift is probably the most reasonable assumption. Drift would be expected to take much longer to produce a significant phenotypic change (Conner & Hartl, 2004), and drift alone is even less likely if the direction of change is generally consistent among populations and if emergence time is controlled by multiple genes (which would be expected to drift independently and not produce a strong directional change). Thus, although genetic drift may have contributed to evolutionary shifts between generations, we consider selection to have been a more likely agent of change.

Whether the observed differences between generations were partially the outcome of adaptive transgenerational plasticity is an open question. Nevertheless, evidence for such “anticipatory” parental effects is weak based on prior studies (Uller, Nakagawa, & English, 2013). Enhanced offspring quality through increased seed mass is one common maternal effect in plants (Roach & Wulff, 1987). However, we controlled for maternal family seed mass effects in our models, and the results of the next‐generation growth chamber experiment confirm that earlier emergence in the drought generation is likely to be a genetic effect. Although we found evidence for reduced variances perhaps due to natural selection, these still may have been influenced by maternally derived epigenetic changes (Germain et al., 2013). For self‐fertilizing plants such as *M. laciniatus*, broad‐sense heritability, which includes maternal effects, is the most relevant agent of adaptive potential (Conner & Hartl, 2004). Thus, although the above effects are conflated in field‐collected seeds, examining first‐generation traits in highly selfing species is potentially as or more important than examining subsequent generation traits for understanding realistic rapid adaptive response. Moreover, our results are consistent with other genetically based adaptive patterns in this species (Sexton et al., 2011), supporting the hypothesis that the observed trait shifts are genetically based and are adaptive under drought. In the Sexton et al. (2011) study, *M. laciniatus* seedling emergence was shown to be under strong natural selection in the field in a fast‐drying, range‐edge environment experiment. A future aim is to understand to what extent epigenetic effects on gene expression or changes in allele frequencies caused the adaptive patterns observed.

Seed quality and longevity is known to decline with seed age (Harrington, 1972), and we attempted to account for this in our study. Seed quality could also affect results if a nonrandom portion of seeds do not germinate, and thus, their correlated traits are not represented; that is to say, the “invisible fraction” effect (Grafen, 1988; Weis, 2018). The cut tests coupled with results from the postsown GA group, in which nearly all cells that were treated germinated within a few days, rule out seed death as important influences in our study. Storage effects may have influenced germination results since emergence was higher in the refreshed generation in growth chambers, although growth chamber conditions were better for all populations (i.e., day length, temperature). To our knowledge, no published study has applied postsown GA treatment as a test of viability. In light of our findings, this technique could be a useful tool for studies diagnosing the above issues or investigating seed banks.

### 4.2 Evidence for climate adaptation

We observed elevation‐based trait differences consistent with climate adaptation, but only in phenological traits. Days to first flower lengthened by elevation, which suggests elevation‐based adaptation by means of flowering time variation (Kooyers, Greenlee, Colicchio, Oh, & Blackman, 2015; Méndez‐Vigo, Picó, Ramiro, Martínez‐Zapater, & Alonso‐Blanco, 2011; Sandring & Ågren, 2009; Stinchcombe et al., 2004). These findings corroborate other studies that have linked phenology to climate adaptation in the yellow monkeyflowers (Friedman & Willis, 2013; Sexton et al., 2011). Although emergence time was observed to decrease significantly with elevation, high‐elevation populations had greatly reduced sample sizes due to more complex germination cues. Moreover, this elevation effect in emergence was lost in the confirmatory generation. Thus, future research is necessary to confirm and understand the significance of this pattern.

Morphological traits did not vary significantly across elevation in the experiment. Fruit mass, total plant mass, and maximum height tended to increase with elevation, but not significantly so (Figure 3). Previous common garden studies have found that plants from lower elevations are often larger, grow more quickly, and flower earlier and for a longer time, while plants from high elevations have the opposite characteristics, which are likely adaptations to conditions at different elevations (Clausen et al., 1941; Conover & Schultz, 1995; Nunez‐Farfan & Schlichting, 2005).

### 4.3 Concluding remarks

One of the foundational studies of local adaptation demonstrated adaptation to elevation in plant populations in the California Sierra Nevada region (Clausen et al., 1941). Since Clausen et al.'s landmark study, we have learned that climate adaptation in plants is very common, although not ubiquitous (Blanquart, Kaltz, Nuismer, Gandon, & Ebert, 2013; Hereford et al., 2009). We have also learned that adaptation can be rapid (e.g., Franks et al., 2007) and that it can involve Mendelian and non‐Mendelian (i.e., epigenetic) inheritance (Feng, Jacobsen, & Reik, 2010; Lynch & Walsh, 1998) of few or many genes (Anderson, Willis, & Mitchell‐Olds, 2011; Bradshaw & Schemske, 2003; Fournier‐Level et al., 2011). However, since Clausen et al., human‐caused climates are changing rapidly, and now, there is great concern about potential range shifts and whether plant species can respond through adaptation.

The finding that there can be a rapid adaptive response to an extreme climate event across the range of a habitat specialist plant contributes to our understanding of plant species distributions and potential persistence under climate change. Resurrecting older genotypes and comparing them to contemporary populations is gaining fast recognition as an important way to empirically test the effects and ramifications of climate change (Franks et al., 2008, 2018), but few studies have done so. We encourage participation in efforts such as Project Baseline (Etterson et al., 2016) and other seed bank programs to facilitate further research.

Climate models predict an increasingly hot and dry future in California, with temperature increases of 1.5–1.8°C by 2,100 and substantial reductions in precipitation (Ackerly, Cornwell, Weiss, Flint, & Flint, 2015; Cayan, Maurer, Dettinger, Tyree, & Hayhoe, 2008). Forecasts also predict a greater proportion of precipitation falling as rain rather than snow (Cayan et al., 2008), which will compress timing of water availability. Thus, there will likely continue to be strong directional selection for traits and phenotypes that correspond with drought tolerance or escape (Etterson & Mazer, 2016; Franks et al., 2007; Jump & Peñuelas, 2005; Schneider & Mazer, 2016). Importantly, although we found evidence for adaptive response, we also found evidence for reduced phenotypic variation. Thus, as climate continues to become hotter and drier, and intense directional selection continues, subsequent reductions in genetic variation (i.e., additive genetic variation) may make adaptation increasingly difficult (Anderson et al., 2012; Jump & Peñuelas, 2005).

Future investigations into selection for adaptive genotypes will be necessary to improve our understanding of climate adaptation in wild systems. Future work could expand to measure performance under various levels of simulated drought and to test for interactive effects of habitat characteristics (i.e., soil type, soil moisture, community composition, etc.). Research is also needed to investigate not only the magnitude of climate stress, but also its duration (i.e., consecutive events) to understand how different populations of a species range can and may respond to stress and selection, including peripheral populations. Finally, research on the specific mechanisms of adaptive response (i.e., gene action, epigenetics, maternal provisioning, etc.) to strong and rapid climate stress in field conditions is greatly needed.

## CONFLICT OF INTEREST

None declared.

## Data Availability

Data from this study have been deposited at Dryad:https://doi.org/10.5061/dryad.h8d2882 (Dickman, Pennington, Franks, & Sexton, 2019).
