# Supplementary material for: Evidence for adaptive responses to historic drought across a native plant species range
Source: Evol Appl. 2019 May 16;12(8):1569–82. doi: 10.1111/eva.12803 (PMC6708426; doi:10.1111/eva.12803)

**Supporting information**

**Table S1.** Study population names, locations and elevations. Latitude and longitude are reported in decimal degrees (DD).

| Population Code | Locality | Latitude (DD*) | Longitude (DD) | Elevation (m) |
| --- | --- | --- | --- | --- |
| R | Zappey Property, Madera County | 37.38136 | -119.66533 | 947 |
| HWY | Highway 168, Fresno County | 37.03977 | -119.40857 | 1000 |
| HH | Poopenaut Valley, Tuolumne County | 37.92159 | -119.81907 | 1020 |
| MC | McLeod Flat, Madera County | 37.35265 | -119.56288 | 1280 |
| HS | Hetchy Sign, Tuolumne County | 37.89389 | -119.84903 | 1400 |
| JM | Jackass Meadow, Madera County | 37.50694 | -119.33867 | 2200 |
| ML | May Lake, Mariposa County | 37.8402 | -119.49213 | 2774 |
| ME | Mammoth Edge, Madera County | 37.69661 | -119.0919 | 3049 |
| HE | Mt. Hilgard, Fresno County | 37.35627 | -118.86088 | 3095 |

**Table S2.** Germination results by population and germination treatment. Original, or untreated, is labeled O. Gibberellic acid treatment is labeled GA. Vernalization treatment is labeled V. Totals (non-italics) and means (italics) are given in the last row.

| Population  (Elevation m) | Seed collection year | Number of maternal families planted | Number of plants that grew-O | Proportion that grew in O germination treatment | Number of plants that grew-GA | Proportion that grew in GA treatment | Number of plants that grew-V | Proportion that grew in V treatment | Total number that grew | Proportion total plants that grew |
| --- | --- | --- | --- | --- | --- | --- | --- | --- | --- | --- |
| R (947) | 2006 | 30 | 14 | 0.47 | 9 | 0.30 | 2 | 0.01 | 25 | 0.83 |
| HWY (1000) | 2005 | 30 | 10 | 0.33 | 10 | 0.33 | 3 | 0.02 | 23 | 0.77 |
| HH (1020) | 2006 | 30 | 6 | 0.20 | 12 | 0.40 | 5 | 0.03 | 23 | 0.77 |
| MC (1280) | 2006 | 30 | 11 | 0.37 | 6 | 0.20 | 3 | 0.02 | 20 | 0.67 |
| HS (1400) | 2005 | 30 | 2 | 0.07 | 14 | 0.47 | 1 | 0.01 | 17 | 0.57 |
| JM (2200) | 2008 | 30 | 1 | 0.03 | 15 | 0.50 | 7 | 0.04 | 23 | 0.77 |
| ML (2774) | 2006 | 30 | 2 | 0.07 | 16 | 0.53 | 4 | 0.02 | 22 | 0.73 |
| ME (3049) | 2006 | 30 | 15 | 0.50 | 7 | 0.23 | 4 | 0.02 | 26 | 0.87 |
| HE (3095) | 2006 | 30 | 2 | 0.07 | 14 | 0.47 | 3 | 0.02 | 19 | 0.63 |
| R (947) | 2014 | 30 | 20 | 0.67 | 6 | 0.20 | 2 | 0.01 | 28 | 0.93 |
| HWY (1000) | 2014 | 30 | 9 | 0.30 | 10 | 0.33 | 1 | 0.01 | 20 | 0.67 |
| HH (1020) | 2014 | 30 | 19 | 0.63 | 4 | 0.13 | 1 | 0.01 | 24 | 0.80 |
| MC (1280) | 2014 | 30 | 23 | 0.77 | 3 | 0.10 | 0 | 0.00 | 26 | 0.87 |
| HS (1400) | 2014 | 30 | 25 | 0.83 | 4 | 0.13 | 1 | 0.01 | 30 | 1.00 |
| JM (2200) | 2014 | 30 | 19 | 0.63 | 5 | 0.17 | 2 | 0.01 | 26 | 0.87 |
| ML (2774) | 2014 | 12 | 2 | 0.17 | 10 | 0.83 | 0 | 0.00 | 12 | 1.00 |
| ME (3049) | 2014 | 18 | 1 | 0.06 | 12 | 0.67 | 1 | 0.01 | 14 | 0.78 |
| HE (3095) | 2014 | 30 | 4 | 0.13 | 12 | 0.40 | 4 | 0.02 | 20 | 0.67 |
| Totals |  | 510 | 185 | *0.36* | 169 | *0.95* | 44 | *0.26* | 398 | *0.78* |

**Table S3.** Levene’s test results for phenology and morphology data. Values in bold were significant at α= 0.05.

**Table S4**. Phenological and morphological traits CV test results.

**Figure S1.** Climatic water deficit (CWD; mm) by elevation and generation. Population names are listed in parentheses.


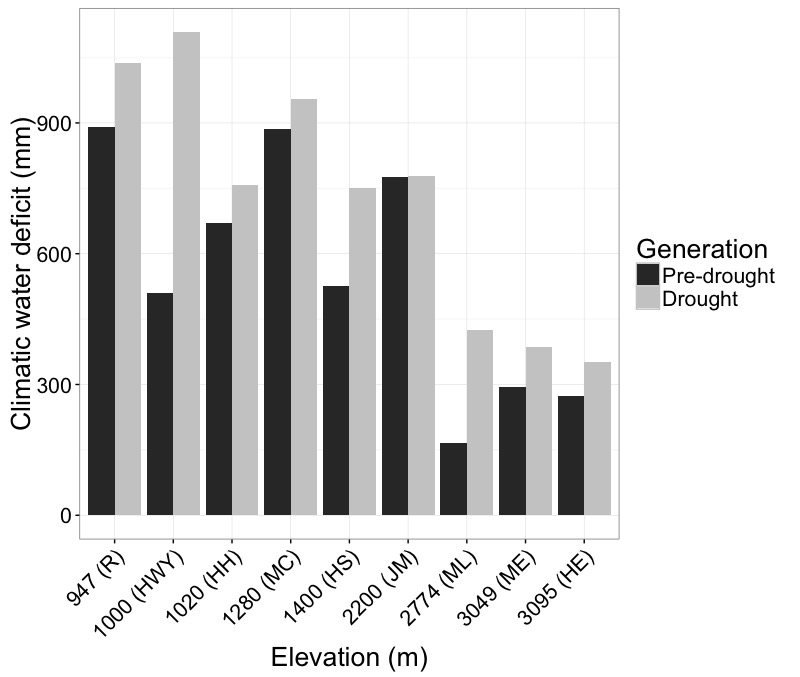

Supplement: Supplementary file 1 [file EVA-12-1569-s001.docx]
